# Cost-effectiveness of birth-dose hepatitis B vaccination among refugee populations in the African region: a series of case studies

**DOI:** 10.1186/s13031-019-0188-y

**Published:** 2019-02-26

**Authors:** Joseph Michael Reardon, Siobhán M. O’Connor, Joseph D. Njau, Eugene K. Lam, Catherine A. Staton, Susan T. Cookson

**Affiliations:** 1 Department of Emergency Medicine, Greenville Health System, 701 Grove Rd, Greenville, SC 29605 USA; 2 Division of Viral Hepatitis, United States Centers for Disease Control and Prevention, 1600 Clifton Rd, Atlanta, GA 30329 USA; 3 Division of Global Migration and Quarantine, United States Centers for Disease Control and Prevention, 1600 Clifton Rd, Atlanta, GA 30329 USA; 4 Division of Global Health Protection, United States Centers for Disease Control and Prevention, 1600 Clifton Rd, Atlanta, GA 30329 USA; 5 Division of Emergency Medicine, Duke University Medical Center, DUMC Box 3096, 2301 Erwin Road, Durham, NC 27701 USA; 6 Emergency Response and Recovery Branch, Division of Global Health Protection, United States Centers for Disease Control and Prevention, 1600 Clifton Rd, Atlanta, GA 30329 USA

**Keywords:** Hepatitis B, refugee, Africa, cost-effectiveness, Djibouti, Algeria, Mauritania, Somalia, Western Sahara, Mali

## Abstract

**Background:**

Hepatitis B affects 257 million people worldwide. Mother-to-child hepatitis B virus (HBV) transmission is a preventable cause of substantial morbidity and mortality and poses greatest risk for developing chronic HBV infection. The World Health Organization recommends that all countries institute universal hepatitis B birth dose (HepB BD) vaccination during the first 24 h of life, followed by timely completion of routine immunization. The objective of this analysis was to assess the cost-effectiveness of adding HepB BD vaccination among sub-Saharan African refugee populations where the host country’s national immunization policy includes HepB BD.

**Methods:**

We performed a cost-effectiveness analysis of three hepatitis B vaccination strategy scenarios for camp-based refugee populations in the African Region (AFR): routine immunization (RI), RI plus universal HepB BD, and RI plus HepB BD only for newborns of hepatitis B surface antigen-positive mothers identified through rapid diagnostic testing (RDT). We focused analyses on refugee populations living in countries that include HepB BD in national immunization schedules: Djibouti, Algeria and Mauritania. We used a decision tree model to estimate costs of vaccination and testing, and costs of life-years lost due to complications of chronic hepatitis B.

**Results:**

Compared with RI alone, addition of HepB BD among displaced Somali refugees in Djibouti camps would save 9807 life-years/year, with an incremental cost-effectiveness ratio (ICER) of 0.15 USD (US dollars) per life-year saved. The RI plus HepB BD strategy among Western Saharan refugees in Algerian camps and Malian refugees in Mauritania camps would save 27,108 life-years/year with an ICER of 0.11 USD and 18,417 life-years/year with an ICER of 0.16 USD, respectively. The RI plus RDT-directed HepB BD was less cost-effective than RI plus delivery of universal HepB BD vaccination or RI alone.

**Conclusions:**

Based on our model, addition of HepB BD vaccination is very cost-effective among three sub-Saharan refugee populations, using relative life-years saved. This analysis shows the potential benefit of implementing HepB BD vaccination among other camp-based refugee populations as more AFR countries introduce national HepB BD policies.

## Background

Hepatitis B virus (HBV) infection is a major cause of preventable morbidity and mortality, affecting 257 million people and causing approximately 887,000 deaths annually worldwide [1]. In nations with high rates of chronic HBV infection, transmission of HBV from mother-to-child poses the greatest risk for developing chronic hepatitis B [2]. Unlike other major infectious diseases, deaths from HBV infection are entirely preventable by low-cost vaccination prior to exposure. Delivery of the hepatitis B (HepB BD) around birth followed by timely completion of the routine hepatitis B vaccine series can prevent 85–95% of HBV transmission, and is recommended by World Health Organization (WHO) [3, 4].

Displaced persons, such as refugees in humanitarian emergencies, are particularly susceptible to HBV transmission because of higher baseline rates of chronic HBV infection, with an average of 7.2% of all refugees having Hepatitis B surface antigen (HBsAg) seropositivity [5]. Establishment of supplemental immunization against rapidly fatal diseases, with routine immunization (RI) established as soon as possible, are among the first interventions advised in refugee situations [6, 7]. No prior studies have examined the cost-effectiveness of HepB BD vaccination among such displaced populations.

WHO recommends that all countries institute universal HepB BD delivery during the first 24 h of life, followed by timely completion of routine immunization [2]. In sub-Saharan Africa (SSA), HepB BD vaccination has been gradually adopted by national Ministries of Health, with WHO targeting universal adoption by 2020 [8]. Three refugee populations are benefitting from HepB BD vaccination in their host countries: Somali refugees in Djibouti, Malian refugees in Mauritania, and Western Saharan refugees in Algeria. In each of these cases, the refugee population did not have access to HepB BD vaccination in the country of origin but their newborns are receiving the HepB BD vaccine due to adoption of a universal HepB BD vaccine policy in the host country [9].

These refugee populations have suffered years of uncertainty. Somali refugees have been displaced to Djibouti for decades, fleeing chronic civil war between the weak central government and separatist insurgents [10]. Somali refugees occupy two major camps in Djibouti, relying on Djibouti’s relative stability compared with its neighbors. Malian refugees have faced a similar struggle, displaced primarily into a single refugee camp in Mauritania after fleeing chronic attacks by disparate desert terrorist organizations that have failed to honor international peace agreements [11]. Refugees from Western Sahara have faced a slightly different situation, having crossed into Algeria to escape decades of conflict between the separatist Polisario movement and the Moroccan government, which claims the territory of Western Sahara [12].

The objective of this analysis was to determine the health impact and cost-effectiveness of RI plus universal HepB BD, or alternatively using a rapid diagnostic test (RDT) for maternal HBsAg to guide HepB BD vaccination, for these three refugee populations in SSA.

## Methods

### Model cohorts and immunization strategies

We examined African countries with existing national HepB BD vaccination policy, and then selected countries hosting large and protracted refugee populations in established camps [13]. We calculated the estimated annual birth cohort for each identified refugee population by multiplying the camp size by the crude birth rate from the country of origin. For each population, we compared life-years lost based on our decision tree model and the cost for each of three strategies for implementation of HepB BD: RI, RI plus universal HepB BD, and RI plus HepB BD provided only for newborns of HBsAg-positive mothers identified through RDT for HBsAg (RDT-directed HepB BD plus RI).

### Model design

We adapted the disease-specific decision-tree models of Lu, et al. and Tu, et al. to estimate the impact and cost-effectiveness of implementing HepB BD by comparing the cost of adding that intervention with life-years lost due to complications of chronic hepatitis B in the refugee camp setting [14, 15]. We constructed the model using TreeAge Pro 2017 (Williamstown, Massachusetts, USA). Variables considered in the analysis are listed in Table 1. The model inputs include mortality due to either HBV-associated cirrhosis or hepatocellular carcinoma (HCC); we did not include fulminant HBV infection at time of acquisition. Additional variables included combined RI and HepB BD vaccine coverage and efficacy, and cost (administrative vaccine purchase and HBsAg RDT). We incorporated population-specific values for vaccine coverage in host country, HBsAg prevalence and fertility rate (but substituting Morocco for Western Sahara due to lack of data), and camp size for refugee camps in Djibouti, Mauritania and Algeria using published figures as shown in Table 1 [10, 12, 16–20]. We did not consider direct medical costs for other complications of hepatitis B because access to medical care is limited in these settings and the likelihood of obtaining prolonged treatment for complications is low.

**Table 1:** Parameter values used in the decision tree model and sensitivity analysis

Parameter	Base case estimate	Sensitivity range	References
Epidemiologic			
Camp size [2015]			
Djibouti [destination from Somalia]	22,080	NA	[10]
Algeria [destination from Sahara]	90,000	NA	[12]
Mauritania [destination from Mali]	48,000	NA	[11]
Crude Birth Rate (births per 1000 per year) [2015]			
Somalia [country of origin]	44	NA	[20]
Western Sahara [region of origin]	20	NA	[20]
Mali [country of origin]	44	NA	[20]
Camp-based Birth cohort per year (Camp size x Crude Birth Rate)			
Djibouti	972	NA	
Algeria	1800	NA	
Mauritania	2112	NA	
Average life expectancy [2015]			
Somalia [country of origin]	56	NA	[29]
Morocco [nearest to Western Sahara]	74	NA	[29]
Mali [country of origin]	58	NA	[29]
Population proportion who are HBsAg+			
Somalia	0.1477	0.1377–0.1584 (95% CI)	[52]
Morocco [substituted for Western Sahara]	0.0109	0.0105–0.0114 (95% CI)	[52]
Mali	0.1307	0.1269–0.1347 (95% CI)	[52]
Transmission rate			
Perinatal transmission in chronic infected mother	0.91	NA	[24]
Disease progression, %			
HBV carrier to chronic infection	0.073	0.003–0.073*	[25]
Chronic hepatitis B to HBV carrier	0.17	0.105–0.306 (Assumed max)	[25]
Chronic hepatitis B to compensated cirrhosis	0.129	0.004–0.153	[53]
Chronic hepatitis B to hepatocellular carcinoma (HCC)	0.005	0.002–0.007	[25]
Compensated cirrhosis to decompensated cirrhosis	0.054	0.028–0.1	[25]
Compensated cirrhosis to HCC	0.03	0.01–0.1	[27]
Mortality rate			
Background mortality	Country-specific tables		[28]
Compensated cirrhosis	0.037	0.03–0.044	[53]
Decompensated cirrhosis	1	0.9–1 (Assumed, − 10%)	Assumed
HCC	1	0.9–1 (Assumed, − 10%)	[53]
Clinical interventions			
Vaccine coverage (Hepatitis B, 3 doses) [2015]			
Djibouti	0.78	0.63–1 (− 25% to 1)*	[15]
Algeria	0.95	0.73–1 (− 25% to 1)*	[15]
Mauritania	0.73	0.55–1 (− 25% to 1)*	[15]
Vaccine protection, %			
Birth dose + Routine Immunization (RI)	0.953	0.946–0.960 (95% CI)	[30]
RI	0.722	0.676–.765 (95% CI)	[30]
Residual Susceptibility in Immunologic Failure	0.05	0.0375–0.0625 (+/− 25%)	[34]
Economic [2015 USD]			
UNICEF price per dose	0.173	NA	[54]
Operational costs per dose or test	0.93	0.11–2.00* (Assumed max)	[26, 44]
Cost per rapid diagnostic test (RDT)	0.50	NA	[55]
Sensitivity of RDT (pooled)	0.948	0.946–0.960	[32]
Specificity of RDT (pooled)	0.995	0.993–1	[32]

*Sensitivity range used in Monte Carlo simulation

Abbreviations: *HBsAg* hepatitis B virus surface antigen, *HBV* hepatitis B virus, *HCC* hepatocellular carcinoma**,** *RDT* rapid diagnostic test

### Disease progression

**Fig. 1:**
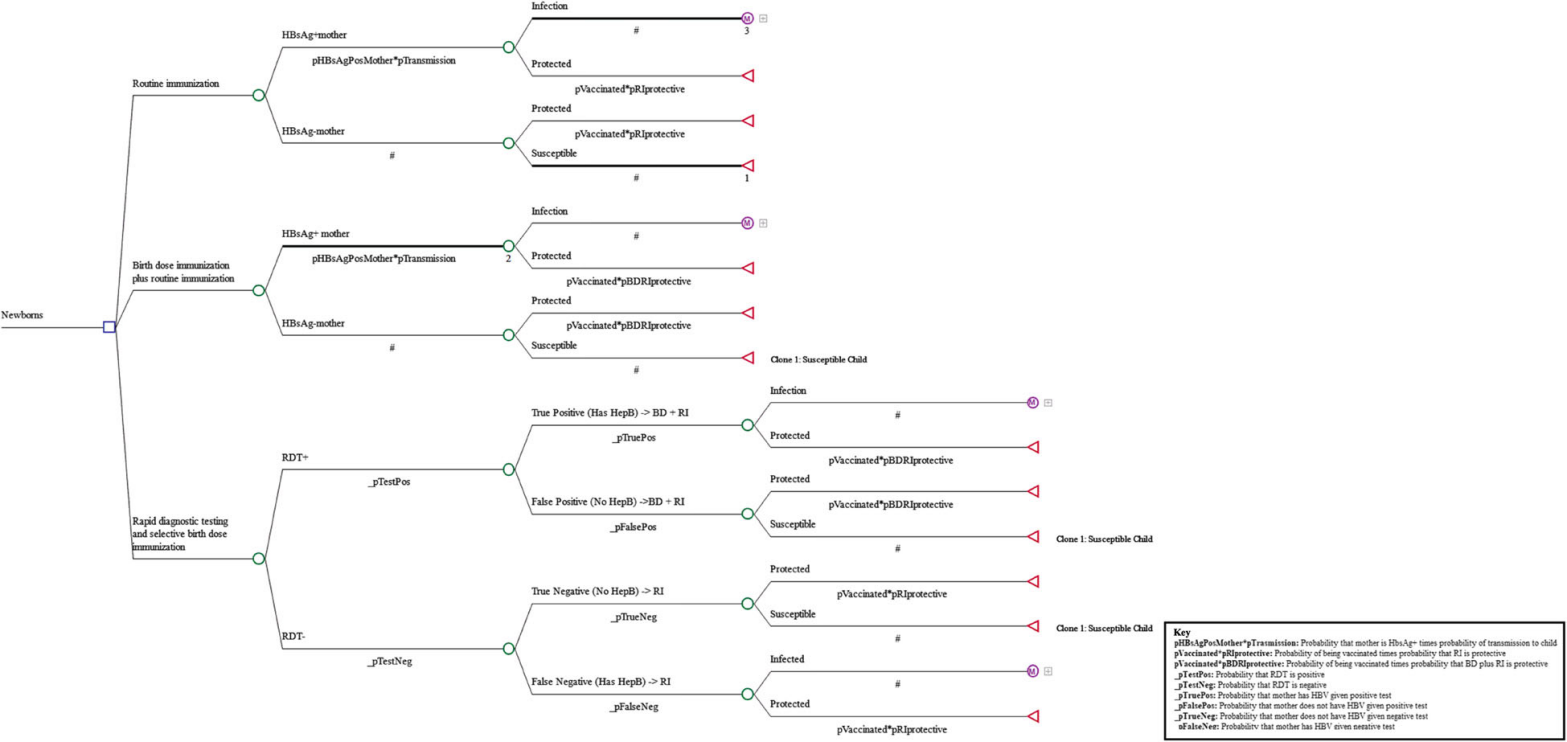
Decision tree modeling probabilities of hepatitis B vertical transmission in three hepatitis B immunization scenarios

**Fig. 2:**
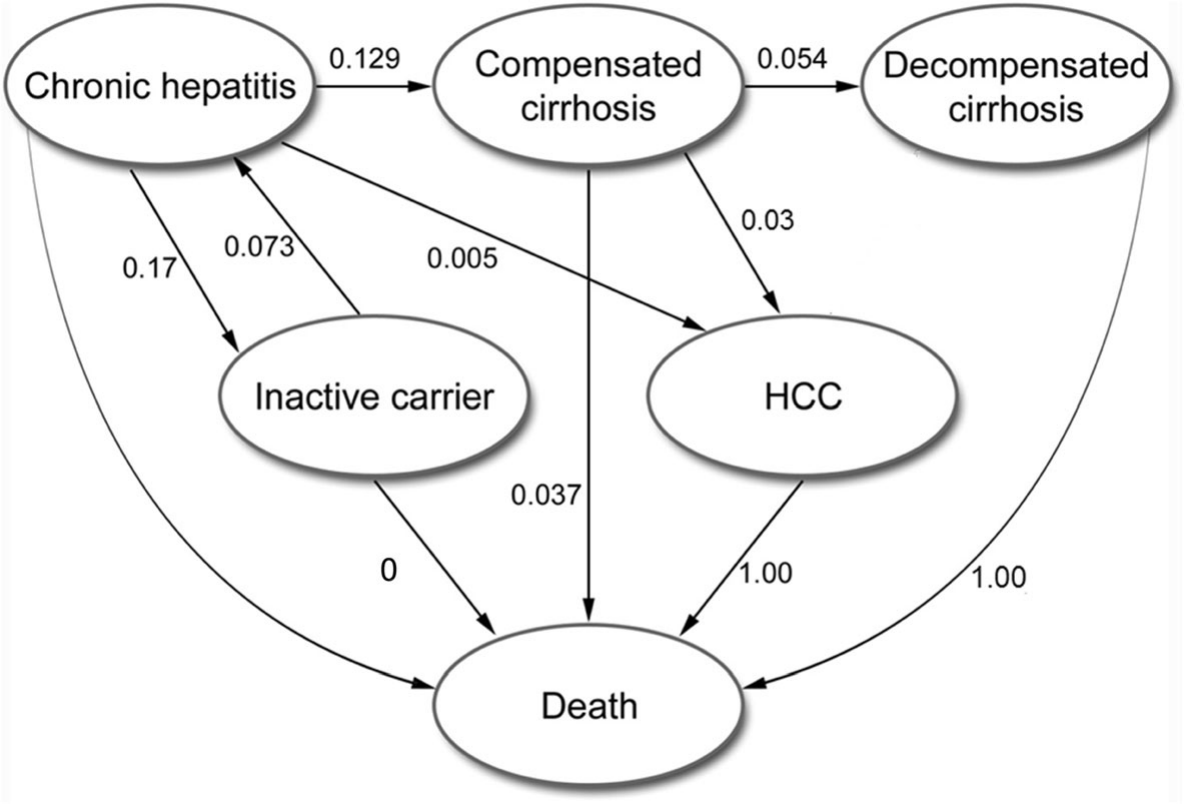
Simplified model of hepatitis B virus infection natural history. Numbers indicate transition probabilities between each state

Our decision tree simulated the vaccination strategies, using maternal HBsAg rate (but substituting Morocco for Western Sahara due to lack of data) as a proxy for HBV prevalence, and calculating probabilities of infant infection based on vaccination coverage and efficacy (Fig. 1). We used a Markov cohort model, cycling yearly, to model hepatitis B progression (Fig. 2). The analytic horizon was from birth to death. We used a global discounting rate of 3% [21]. We modeled four states, with HBV inactive carrier status and chronic active hepatitis B moving between each other at fixed probabilities, chronic hepatitis capable of progressing to compensated cirrhosis or HCC, and compensated cirrhosis able to progress to decompensated cirrhosis or HCC. Chronic hepatitis is defined as persistent hepatic inflammation, whereas cirrhosis is defined as liver fibrosis affecting the liver’s synthetic functioning [22]. Compensated cirrhosis is defined as fibrosis with liver dysfunction that does not substantially impair the individual’s daily activity, whereas decompensated cirrhosis encompasses gastrointestinal bleeding, encephalopathy and massive ascites that are clinically apparent and usually fatal in the short term [23]. Transition probabilities of movement from one state to another during hepatitis B infection were based on published literature [24–27].

### Input parameters

For birth rate, life expectancy and HBSAg prevalence, we used the country of origin of the refugees, except for life expectancy from Western Sahara because the measure was not available; the estimate for Morocco was substituted. We obtained life expectancies from the World Bank and age-specific background mortality rates and vaccine coverage data from WHO tables [16, 28, 29].

To approximate the vaccine efficacy of RI plus universal HepB BD versus RI alone, we applied figures from a retrospective study of vaccine efficacy for preventing vertical transmission of HBV infection in Canada, comparing HBsAg prevalence rates among children who were vaccinated within 3 days of birth to those who were vaccinated more than 8 days after birth [30].

### Cost

We used UNICEF prices for multi-dose monovalent hepatitis B vaccine (HepB BD), and program costs (vaccine purchase, injection supplies, vaccine distribution and service delivery) from a similar study in the Gambia, updating for inflation [26, 31]. We chose cost of the highly sensitive and specific Alere Determine™ HBsAg test (Waltham, MA), widely available in Africa, to represent HBsAg RDT [32].

We assumed RI costs in all countries were from government perspective. Costs of implementing HepB BD delivery were therefore calculated from the public payer perspective (i.e., the cost to the government to procure and deliver the vaccine).

### Model Assumptions

Our model has several key assumptions.

On the public health level, we assumed that infants would receive HepB BD vaccination at the same rate as RI and all receiving HepB BD would go on to receive RI, as they receive health services within the camp setting. We varied this assumption by a margin of - 25% of estimate to 100% in the sensitivity analysis [33]. We assumed that the operational costs for performing RDT were similar to the operational costs for delivery of HepB BD vaccine as they both require trained fieldworkers, supplies and transport, and we varied this assumption in the sensitivity analysis as exact operational costs for RDT are not available. We assumed that external stressors are approximately equal between camps as all refugee camps in this analysis are supervised by the United Nations High Commissioner for Refugees. We used a residual susceptibility rate of 5% for likelihood of acquiring hepatitis B horizontally in the event of immunologic failure [34].

For disease-specific variables, we made several simplifying assumptions about disease progression to facilitate demonstration of the model. We assumed that all mother-to-child HBV transmission happened in the perinatal period, as prior studies have shown that later vertical transmission is less than or equal to 1% [35, 36]. We did not calculate disability-adjusted life years (DALYs) lost or incidental costs to families or communities as the progression from fulminant disease (decompensated cirrhosis or HCC) to death is likely rapid (within one year) in the absence of dedicated health services as shown in the literature [37, 38]. A recent study supported this assumption, finding that globally, years of life lost due to hepatitis make up 98% of the burden of the disease encapsulated by disability-adjusted life years (DALYs) [39].

We assumed that infants did not die from fulminant hepatitis B, as the rate of death from perinatal hepatitis is low (less than 1 in 1000) [40]. We used overall HBV transmission rates from an HBV-infected mother (both hepatitis B e antigen-positive and -negative) to her child from the literature [24]. We also did not include the effect of co-infection with HIV as reported HIV rates in the studied areas are low enough (0.1 to 1.6%) to not significantly alter the results of our model [41].

### Outcome measures

We compared number of life-years lost from HBV attributable HCC- and cirrhosis-related premature death among the RI, RI plus universal HepB BD, and RDT-directed HepB BD plus RI scenarios and calculated cost per life year saved and incremental cost-effectiveness ratio (ICER) under each scenario.

### Parameter uncertainty

We conducted one-way sensitivity analyses, varying the parameters one by one with the range of values shown in Table 1. To estimate the prevalence of HBsAg prevalence in refugee populations, we used sources from the published literature [42] and examined sensitivity around these values.

To determine the sensitivity around estimates of stochastic variables, we performed a second-order Monte Carlo simulation with 1000 iterations. We varied vaccination coverage, probability of HBV carriers transitioning to chronic hepatitis, cirrhosis and HCC, and the cost of operations according to triangular distributions, with the lower and upper bounds of vaccination coverage representing the worst and best performing countries in the African region [43]. We varied the transition to complications probability bounds by 25%, and varied the operational cost, with the lower bound representing the lowest estimate for the percentage of operational cost relative to vaccine cost determined by the Expanded Program on Immunization Costing study [44], and the upper bound of operational cost varied upward to 2 USD to account for vaccine deployment during rainy season or extreme condition. We assumed a fixed cost for the vaccine and RDT.

## Results

### Impact of vaccination

The impact of HepB BD vaccination was similar across multiple camp situations; it would save 506 life-years per 10,000 refugees per year among refugees in Djibouti, 126 life-years per 10,000 refugees per year among refugees in Algeria, and 2204 life-years per 10,000 refugees per year among refugees in Mauritania (Table 2).

**Table 2:** Health and cost outcomes of one-year refugee birth cohorts under different hepatitis B vaccine immunization scenarios for refugees resettled from Western Sahara to Algeria, Somalia to Djibouti and Mali to Mauritania [24, 41, 54, 55]

Strategy	Relative Life-Years Saved per Camp	Life-Years Saved per 10,000 Refugees	Vaccine and Program Costs (USD) per Camp	Incremental Cost (USD) per Camp
Djibouti				
Routine immunization	–		3460	–
Birth dose plus routine immunization	1118	506	4393	933
Rapid diagnostic testing plus birth dose and routine immunization	807	365	4471	1011
Algeria				
Routine immunization	–		6408	–
Birth dose plus routine immunization	1134	126	8208	1800
Rapid diagnostic testing plus birth dose and routine immunization	180	20	8172	1764
Mauritania				
Routine immunization	–		7519	–
Birth dose plus routine immunization	10,581	2204	9420	1901
Rapid diagnostic testing plus birth dose and routine immunization	9905	2064	9567	2049

Abbreviation: *USD* United States dollars

### Cost-effectiveness

The Monte Carlo simulations showed a trend toward increased effectiveness as cost increased, but a relative ceiling of effectiveness was seen despite increasing cost when the proportion of children receiving RI approached 1.

In each case, the cost per year of life saved by addition of HepB BD is much less than the average gross domestic product (GDP) of the host country (0.83 USD versus 3342 USD in Djibouti, 1.59 USD versus 14,613 USD in Algeria and 0.18 USD versus 3835 USD in Mauritania) [45]. In all countries, RDT-directed HepB plus RI was less cost-effective than RI alone or RI plus universal HepB BD.

### Sensitivity analysis

**Fig. 3:**
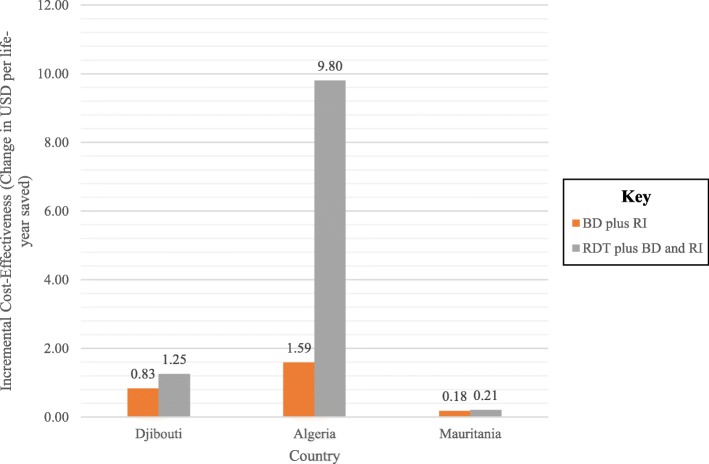
Incremental cost-effectiveness ratios of adding hepatitis B vaccine birth dose (HepB BD) to routine immunization (RI) or RI plus HepB BD delivery only to newborns of HBV-infected mothers diagnosed by rapid diagnostic testing for hepatitis B surface antigen, compared to RI alone among camp-based refugees resettled from Western Sahara to Algeria, from Somalia to Djibouti and from Mali to Mauritania

**Fig. 4:**
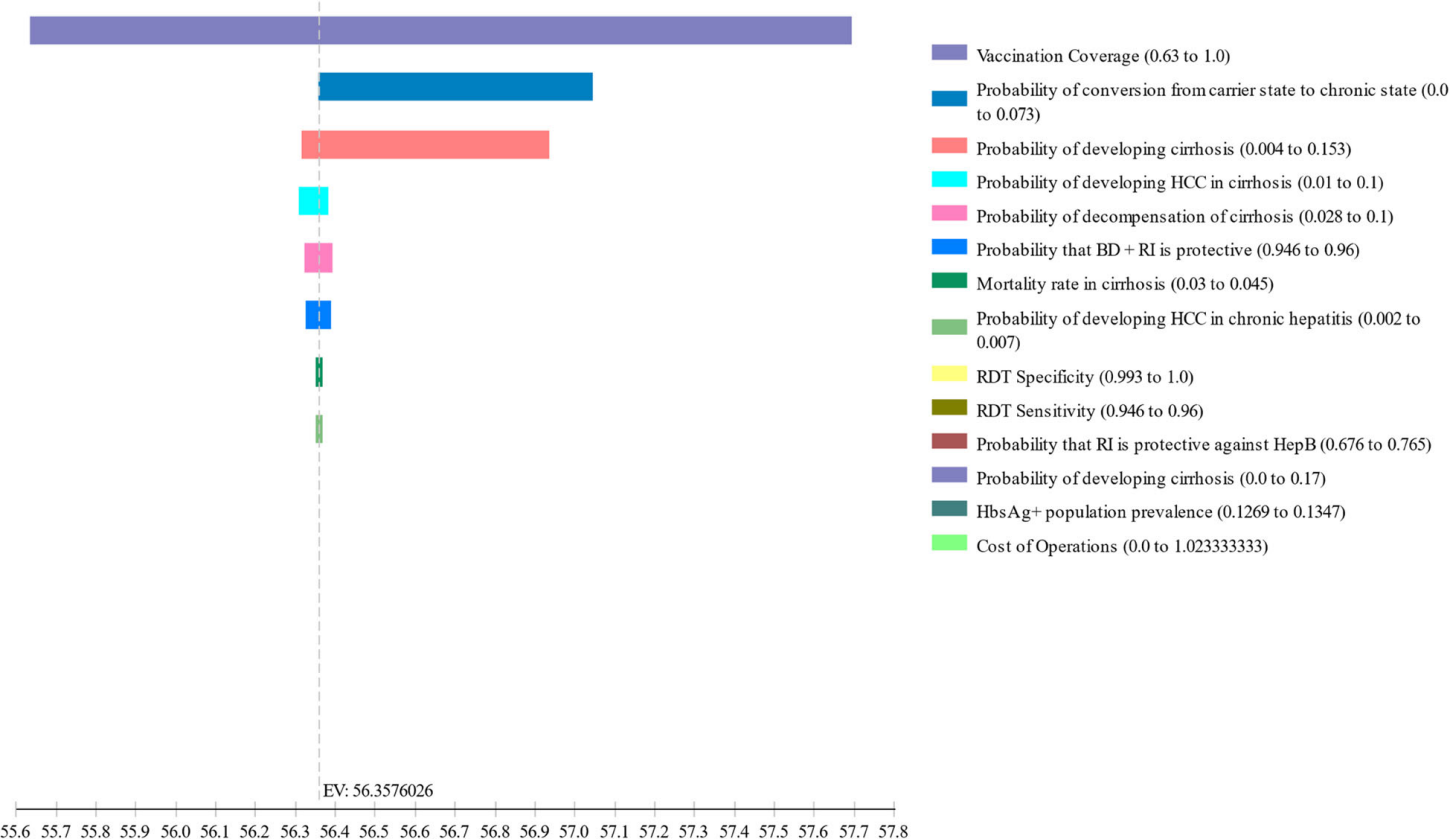
Tornado sensitivity analysis demonstrating input variables affecting the net benefits of hepatitis B vaccine birth dose delivery followed by routine immunization versus routine immunization alone Malian refugees relocated to refugee camps in Mauritania

Using triangular probability distributions, we found that none of the confidence intervals around transition probabilities in the Markov model affected the final cost, effectiveness, or ICER by more than 2% in any analysis. In sensitivity analyses, the baseline vaccination rate had the greatest impact on net health benefit. For each country, net life years gained varied across a range of 2.1 to 2.4 years when considering a − 25% of estimate to 100% in baseline vaccination rate. Variance in protection of RI plus universal HepB BD against HBV infection was the second greatest contributor to uncertainty in net benefits, given the uncertainty of protection from prior studies [30]. The ICER was more favorable for RI plus universal HepB BD than for a test and treat strategy (Fig. 3). The incremental cost-effectiveness of RI plus universal HepB BD over RI alone was most affected by the probability that RI is protective against hepatitis B. The greatest variability in net benefits upon sensitivity analysis was seen in the Mauritanian refugee population (Fig. 4). Sensitivity analyses were similar for all countries.

In the Monte Carlo simulation, we observed overlap in both cost and effectiveness for all three approaches, although RI plus universal HepB BD was both more effective and more cost-effective in terms of dollars spent per year of life saved than either RI alone or RDT-directed HepB plus RI under most scenarios. All approaches remained very cost-effective under all simulated scenarios.

## Discussion

The combination of RI plus universal HepB BD was very cost-effective for the three refugee populations analyzed, based on the criterion that an intervention must cost less than the annual GDP per capita of the region of interest [46]. Each of the host countries studied has begun to implement universal HepB BD vaccination [9], and our results show that this strategy should provide considerable benefit to the refugee populations of these regions, with a net 55,332 life years saved per year. However, the total life-years saved per 10,000 refugees for Somalian and Malian refugees were higher than the total for Western Saharan refugees, reflecting those countries’ larger prevalence of HBsAg positive mothers. Use of RDT-directed HepB plus RI was also found to be cost-effective, but the savings were inferior to RI plus universal HepB BD vaccination both in incremental cost-effectiveness and in relative life-years saved.

HepB BD vaccination has been found to be very cost-effective in nearly every other situation in which it has been studied, including in low-resource and high-resource settings [15, 26, 47]. Cost-effectiveness in prior studies was only reduced in situations in which baseline maternal HBV prevalence was very low, which is unlikely in these refugee camps given the nearly universally high prevalence of HBsAg seropositivity reported across refugee populations [5, 48]. However, refugee camps often present a different set of health priorities and more pressing needs, such as water, food or safety may temporarily overshadow vaccination, especially with new and underutilized vaccines [6]. Our results suggest that HepB BD vaccination must remain among the top priorities in refugee camps.

Refugee camps also represent a unique situation for global health, as some refugees are in the process of being resettled to a third asylum country. In 2014 alone, over 73,000 refugees were resettled, the majority of them moving to North America, Australia and the Nordic countries [49]. The program cost calculated here for all of the target refugee camps is less than the cost for treatment of a single episode of decompensated cirrhosis or HCC in a wealthier country. Our findings highlight that investment in health services in refugee camps and host communities themselves are likely to yield great benefits at low cost.

Our sensitivity analysis shows that the two main factors affecting the incremental cost-effectiveness of HepB BD vaccination are vaccine coverage and vaccine efficacy. Data from early clinical trials of HepB BD vaccination show substantial improved protection and it would be unethical to conduct further randomized trials [4, 24]. The only semi-randomized study demonstrating vaccine efficacy was under-powered and limited by methodological limitations [3]. Improving HepB BD coverage rates is the best way to maximize benefit from this intervention.

Global increases in vaccination have been one of the great success stories of the modern era. The advent of monitoring and reporting of vaccination rates has been coupled with steady improvement in vaccination rates in almost every country [33, 43]. Other means of improving vaccine uptake, including market-based approaches and multilateral partnerships, have been considered but inconsistently applied [50]. We expect that the advent of reporting of HepB BD vaccination rates in refugee camps along with concomitant health systems strengthening would catalyze continued improvements. This health system strengthening among a displaced population will not only benefit the refugees but also add to overall global health security.

The cost-effectiveness modeling used here employs existing data in the literature on both the natural history of perinatal-transmitted HBV and current vaccination costs. Such modeling can be easily and flexibly applied to other new and underutilized vaccines such as rotavirus, rubella, typhoid fever, rabies, and human papillomavirus in order to help create an evidence base for prioritizing each intervention in refugee settings [51].

### Limitations

This analysis has several limitations. Firstly, our probability point estimates for disease modelling and cost were drawn from existing literature and may not necessarily reflect the populations under study. We used a static model that does not incorporate horizontal transmission of HBV, which limits our ability to calculate life-years saved through protection from post-perinatal transmission. However, the benefits of RI to protect against horizontal transmission of HBV are already well-known [52]. We assumed that all refugees receiving HepB BD vaccination would receive RI, which may be interrupted in unstable situations. It is also possible that refugees may not accept vaccination due to cultural reasons or the perception that complications of hepatitis are too far in the future to warrant preventive measures now. We attempted to account for this possibility through the sensitivity analysis around actual vaccination rates in our model. [33], which is why we used the lower of the two estimates of vaccine coverage in order to generate a more conservative model.

We made several simplifying assumptions about the natural history of HBV infection, including a 100% one-year mortality rate for patients diagnosed with HCC and presuming that HCC is non-treatable [53]. The survival rates of patients with decompensated cirrhosis and HCC are unknown among populations with limited access to medical care. We did not include disability adjustment because we assume that there is rapid progression to death in the refugee population.

## Conclusions

HepB BD vaccination is very cost-effective across three SSA refugee populations. Regular reporting of BD vaccination rates by humanitarian agencies will help to demonstrate the benefits accrued. This analysis shows the benefit of HepB BD vaccination among camp-based refugee populations and should be implemented in other refugee camps as other more AFR countries introduce national HepB BD policies by 2020 and thereby add to strengthen the overall global health security among these mobile, vulnerable populations [54].

## Abbreviations

AFR: African Region
HBsAg: hepatitis B virus surface antigen
HBV: hepatitis B virus
HCC: hepatocellular carcinoma
Hep B BD: hepatitis B vaccine birth dose
HIV: human immunodeficiency virus
ICER: incremental cost-effectiveness ratio
LY: life year
RDT: rapid diagnostic test
RI: routine immunization
SSA: Sub-Saharan Africa
UNICEF: United Nations Children’s Fund
USD: United States dollars
WHO: World Health Organization
